# Pregnancy Outcomes in Women with PCOS: Follow-Up Study of a Randomized Controlled Three-Component Lifestyle Intervention

**DOI:** 10.3390/jcm12020426

**Published:** 2023-01-05

**Authors:** Alexandra Dietz de Loos, Geranne Jiskoot, Yvonne Louwers, Annemerle Beerthuizen, Jan Busschbach, Joop Laven

**Affiliations:** 1 Division of Reproductive Endocrinology and Infertility, Department of Obstetrics and Gynaecology, Erasmus University Medical Center, 3015 GD Rotterdam, The Netherlands; 2 Department of Psychiatry, Section Medical Psychology and Psychotherapy, Erasmus University Medical Center, 3015 GD Rotterdam, The Netherlands

**Keywords:** polycystic ovary syndrome, PCOS, obesity, conception, live birth, lifestyle intervention, multi-component

## Abstract

Women with polycystic ovary syndrome (PCOS) and excess weight often present with reproductive derangements. The first-line treatment for this population is a multi-component lifestyle intervention. This follow-up study of a randomized controlled trial based on data from the Dutch Perinatal registry was conducted to study the effect of a one-year three-component (cognitive behavioral therapy, healthy diet, and exercise) lifestyle intervention on pregnancy outcomes in women with PCOS and overweight or obesity. Women diagnosed with PCOS, a BMI ≥ 25 kg/m², and a wish to conceive were randomized to either three-component lifestyle intervention (LSI, n = 123), and care as usual (CAU, n = 60) where they were encouraged to lose weight autonomously. Conception resulting in live birth was 39.8% (49/123) within LSI and 38.3% (23/60) within CAU (*p* = 0.845). In total, 58.3% conceived spontaneously. Gestational diabetes (LSI: 8.2% vs. CAU: 21.7%, *p* = 0.133), hypertensive disorders (LSI: 8.2% vs. CAU 13.0%, *p* = 0.673), and preterm birth (LSI: 12.2% vs. CAU: 17.4%, *p* = 0.716) rates were all lower in LSI compared to CAU. This follow-up study showed no significant differences in conception resulting in live birth rates between LSI and CAU. Nonetheless, a large proportion eventually conceived spontaneously. Moreover, after LSI, the number of uneventful pregnancies was lower compared to care as usual.

## 1. Introduction

Polycystic ovary syndrome (PCOS) is the most common endocrine disorder in women of reproductive age, and is defined by the presence of at least two of the following key characteristics according to the Rotterdam 2003 criteria: ovulatory dysfunction, hyperandrogenism, and polycystic ovarian morphology [1,2]. Moreover, PCOS is associated with overweight and obesity [3], and excess weight is known to have a positive correlation with the PCOS phenotypical severity status [4]. Overall, women with PCOS and overweight or obesity present with more pronounced clinical, metabolic, and reproductive derangements [5,6,7].

Reproductive problems in women with PCOS generally present as irregular or absent menstrual cycles (oligo- or amenorrhea respectively), which are signs of anovulatory subfertility. The ovulation rate is negatively affected by obesity, resulting in lower chances of spontaneous pregnancy [8]. Obesity also causes inferior outcomes with regard to infertility treatments when compared to women with a normal weight [9,10]. Moreover, when pregnant, complications such as gestational diabetes, hypertensive disorders, preterm birth, and stillbirth seem to be more prevalent in this population [11,12,13,14,15]. Hence, a wish to become pregnant is not so self-evident for women with PCOS, especially if they are overweight or obese.

The current first-line treatment for women with PCOS is a multicomponent lifestyle intervention (diet, exercise, behavioral therapies) in order to lose weight and to prevent excess weight gain [1]. Despite pregnancy not being the primary aim of many studies, some lifestyle intervention trials have reported on incidental pregnancy findings [16,17]. Nonetheless, a recent meta-analysis investigated reproductive outcomes after lifestyle interventions compared to minimal treatment in women with PCOS and concluded that there are no lifestyle studies available with live birth as a primary outcome [18]. Hence, the international PCOS guideline highlighted the critical need for more research with regard to pregnancy outcomes following lifestyle interventions [1].

In line with this PCOS guideline, we performed a randomized controlled long-term three-component lifestyle intervention, with or without additional short message service (SMS) support, in overweight or obese women with PCOS. Previous results on the primary outcome measure of weight loss demonstrated that our three-component lifestyle intervention program resulted in reasonable weight loss in women with PCOS, and adding SMS resulted in even more weight loss [19]. The aim of the current follow-up study was to evaluate conception resulting in live birth rates within 24 months after the start of the lifestyle intervention (LSI) compared to care as usual (CAU). Furthermore, time to conception after the start of the intervention, mode of conception, pregnancy complications, and neonatal outcomes were also evaluated. We hypothesized that pre-pregnancy weight loss and the adoption of a healthy lifestyle would cause more pregnancies, shorter time to conception, and less pregnancy complications.

## 2. Materials and Methods

### 2.1. Trial Design

This was a follow-up study from a randomized controlled trial (RCT) based on data from the Dutch Perinatal registry. The timeframe for data collection from the Dutch Perinatal registry per participant comprised a total of 24 months after the start of the study (0–12 months (during study period) and 12–24 months (post-study period)). The RCT was a one-year three-component lifestyle intervention study which was performed between August 2010 and March 2016. Three groups were compared: one-year lifestyle intervention with additional SMS support (SMS+), one-year lifestyle intervention without additional SMS support (SMS−), and one-year care as usual (CAU). We have previously published the study protocol [20]. For the current follow-up study, we combined the SMS+ and SMS− groups into one lifestyle intervention group (LSI). This RCT was approved by the Medical Research Ethics Committee of the Erasmus MC in Rotterdam (MEC 2008-337) and registered by clinical trial number: NTR2450 (www.trialsearch.who.int, accessed on 2 August 2010).

### 2.2. Participants

Women were included within the division of Reproductive Endocrinology and Infertility of the Department of Obstetrics and Gynaecology, at the Erasmus MC, the Netherlands, when they were actively trying to get pregnant, had a body mass index (BMI) > 25 kg/m², were between 18–38 years of age, and had a diagnosis of PCOS according to the Rotterdam 2003 consensus criteria [2]. Women were excluded when they had inadequate command of the Dutch language, severe mental illness, obesity due to another somatic cause, androgen excess caused by adrenal diseases or ovarian tumours, and other malformations of the internal genitalia.

The sample size calculation of the RCT was based on a notable difference in weight as the primary outcome measure. All participants provided written informed consent. Subsequently, participants were randomly assigned in a 1:1:1 ratio to one of the three groups of the study with the use of a computer-generated random numbers table. This procedure was executed by a research nurse who was not involved in the study. Assignment was made by sequentially numbered, identical, sealed envelopes, each containing a letter designating the allocation [20].

### 2.3. Three-Component Lifestyle Intervention (LSI) and Control Group (CAU)

The lifestyle intervention covered three main components during twenty 2.5 h group meetings over the period of one-year: (1) normo-caloric diet, as recommended by the “Dutch Food Guide” [21], (2) exercise according to the “Global Recommendations for physical activity by the World Health Organization” [22], (3) cognitive behavioral therapy, in order to create awareness and to restructure dysfunctional thoughts about, e.g., self-esteem and weight (loss). After three months the SMS+ group were sent weekly self-monitored information regarding their diet, physical activity, and emotions by SMS, and received patient-tailored SMS feedback by a semi-automated software program in order to provide social support and to encourage positive behavior. The LSI was first tested in a pilot group (n = 26) in order to get acquainted with the program and procedures. These data were not used for the study.

The control group received care as usual over the period of one year. The risk of excess weight for both mother and child, and the relation between overweight and infertility was discussed by their treating physician. Subsequently, weight loss was encouraged by publicly available services such as visiting a dietician or gym.

Participants in both groups (LSI and CAU) had a wish to become pregnant. They were encouraged to lose 5–10% of their initial body weight as their personal goal during the course of the study. Provided that they could sustain their weight loss for at least three months and complete the one-year study, participants received assisted reproductive care. In the meantime, spontaneous pregnancies could also occur during the one-year study and in the one-year follow-up period after the study. Participants did not receive further interventions if they became pregnant spontaneously during the course of the study.

### 2.4. Clinical and Endocrine Assessments

All participants received five standardized assessments from baseline till one year. These included general medical, obstetric and family history, and physical measurements (height, weight, BMI (kg/m²), waist and hip circumference, and blood pressure). In addition, a transvaginal ultrasound (probe < 8 MHz) was performed and fasting blood samples were collected for an extensive endocrine assessment.

Pregnancy and neonatal outcomes were collected from the Dutch Central Bureau for Statistics (CBS) combined with the Dutch Perinatal registry (Perined). Maternal, neonatal and delivery characteristics are routinely registered by caregivers (midwives, gynecologists, and pediatricians) using electronic registration forms which are all collected by the Perined registry. This results in available population based data on approximately 96% of all deliveries and pregnancies in the Netherlands [23]. Information on miscarriages or deliveries < 16 weeks of gestational age is not available. Data from all participants were linked to the Perined registry by the Dutch CBS using pseudo-anonymization.

### 2.5. Outcome Measures

The primary outcome measure of the current follow-up study was conception within 24 months after the start of the intervention resulting in live birth. Live birth was defined as the delivery of a living child. Secondary outcome measures included time to conception (from start intervention until conception), mode of conception (spontaneous or by assisted reproductive technology (ART)), pregnancy complications such as (gestational) diabetes, hypertensive disorders (hypertension and/or (pre) eclampsia), and preterm birth (birth <37 months of gestational age). Other secondary outcome measures included neonatal outcomes and complications such as neonatal intensive care unit (NICU) admission, small for gestational age (SGA) (birth weight < 10th percentile), large for gestational age (LGA) (birth weight > 90th percentile) and congenital abnormalities.

### 2.6. Statistical Methods

Data were analyzed according the intention-to-treat principle. Outcome measures were displayed as n (%) or median (interquartile range (IQR)). Differences between the groups (LSI (SMS+ and SMS− combined) vs CAU) were tested with the χ^2^ test or Fishers exact test for categorical variables and with the Mann–Whitney U test for continuous outcomes.

A survival analysis was performed to calculate time to conception and differences between the groups were tested with the log rank test. Logistic regression analyses were used to evaluate the association between changes in weight within the groups and the chance to get pregnant.

Finally, different baseline characteristics were evaluated as predictors for conception within 24 months after the start of the intervention. These baseline characteristics were selected as potential predictors based on a literature search and included: study group, age, BMI, modified Ferriman–Gallwey score (mFG), waist circumference, time attempting to conceive before the start of the study, prior parity, smoking, testosterone, androstenedione, free androgen index (FAI), glucose, insulin, sex hormone-binding protein (SHBG), luteinizing hormone (LH), follicle stimulating hormone (FSH), estradiol, mean ovarian volume, mean ovarian follicle number, and menstrual cycle. Logistic regression analyses were used for the analyses of these potential predictors on conception. First, with univariate models we identified predictors with a significance of *p* < 0.200. Second, these identified potential predictors were entered in a multivariate model following a stepwise elimination of the least significant predictor until the final remaining variables reached a significance of *p* < 0.05. Outcomes were displayed as odds ratio (OR) with 95% confidence interval (CI). All models were corrected by including baseline weight as a covariate. Analyses were performed with IBM SPSS statistics version 25.0.

## 3. Results

**Figure 1:**
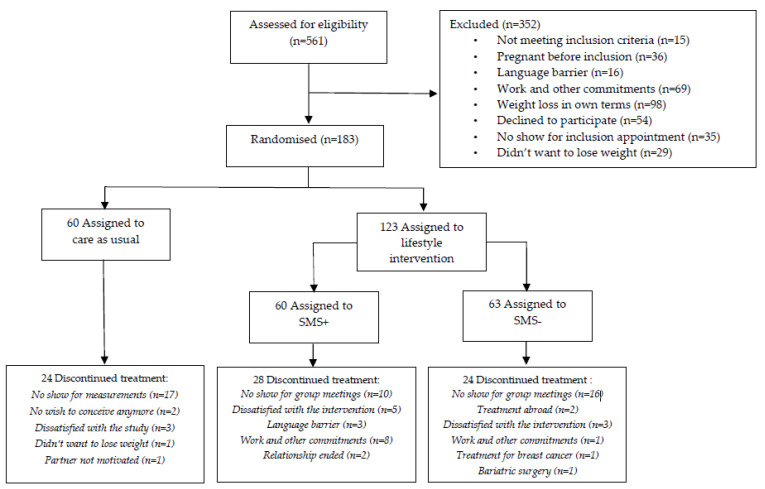
CONSORT flowchart.

A total of 561 women were eligible for the trial between 2 August 2010 and 11 March 2016. Figure 1 shows the participation selection flow-chart. To summarize, 26 women were included in the pilot study; 352 women could not participate because of various reasons; and finally 183 women were randomly assigned to one of the three arms of the study: (1) SMS+ group (n = 60), (2) SMS− group (n = 63); resulting in a total of n = 123 women in the LSI group, and (3) CAU group (n = 60). Baseline characteristics were presented in Table 1. Median age was 29 years (26–32)for LSI and 28 years (26–32) for CAU. BMI at baseline was 33.6 (30.8–36.6) for LSI and 30.6 (29.3–34.3) for CAU. Time attempting to conceive before the start of the study was 24 (15–38) and 23 (14–35) months for the LSI and CAU groups, respectively. The majority of the participants were nulliparous with 77.7% in LSI and 75.9% in CAU. Our previous results from this RCT demonstrated a statistically significant (*p* < 0.001) within-group mean weight loss of 7.87 kg in SMS+, 4.65 kg in SMS− and 2.32 kg in CAU after one year [19]. The following pregnancy results are based on calculations by the Erasmus MC using non-public microdata from Statistics Netherlands.

### 3.1. Conception Resulting in Live Birth

**Figure 2:**
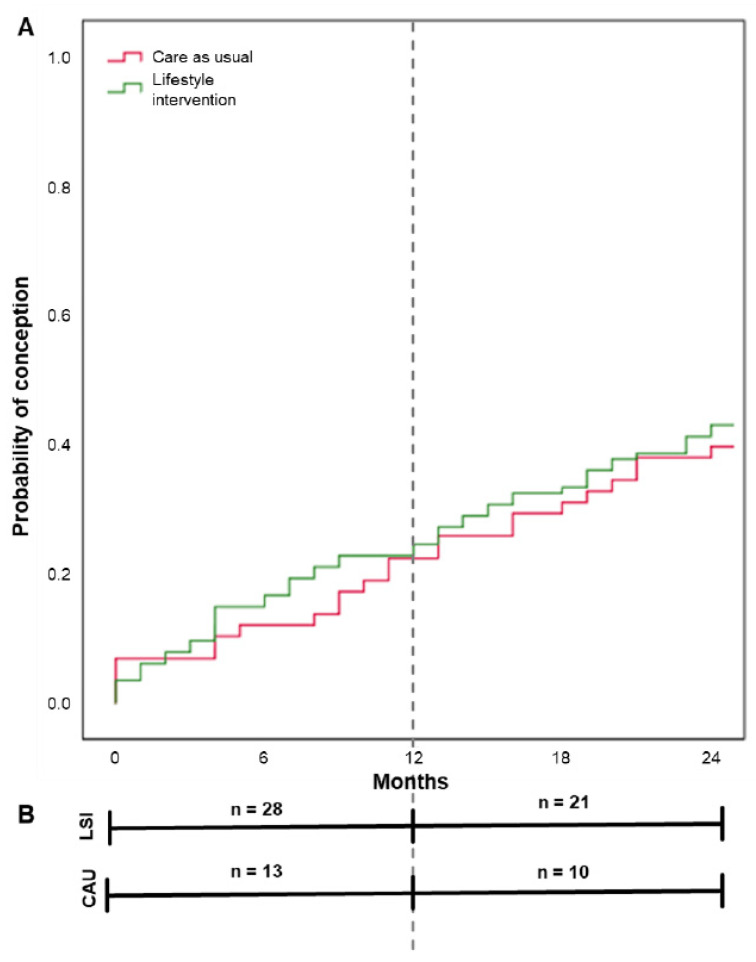
Time from the start of the study to conception resulting in live birth by group. Note: Results are based on calculations by the Erasmus MC using non-public microdata from Statistics Netherlands. (**A**) shows the Kaplan–Meier curve with mean time to conception resulting in live birth for lifestyle intervention (18.7 months), and care as usual (19.4 months). Differences were tested with the log rank test (*p* = 0.646). (**B**) shows the number of conceptions resulting in live birth within the given timeframe 0–12 months (during study period) and 12–24 months (post-study period) per study group.

**Figure 3:**
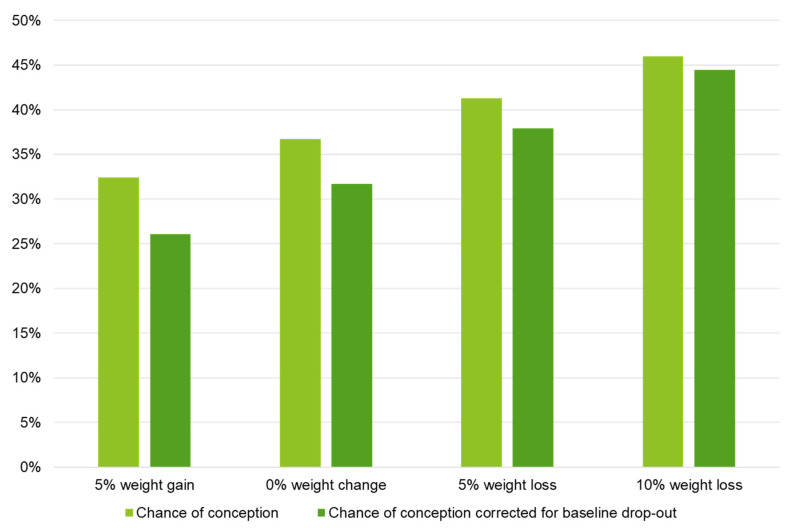
Logistic regression model for the effect of changes in weight on the chance of conception ≤ 24 months after the start of the intervention resulting in live birth. Note: Results are based on calculations by the Erasmus MC using non-public microdata from Statistics Netherlands. Logistic regression analyses; chance of conception: B = −0.038 SE 0.028, *p* = 0.169; chance of conception corrected for baseline drop-out: B = −0.055 SE 0.031, *p* = 0.081.

Within 24 months after the start of the intervention, the conception resulting in live birth rate was 39.8% (49/123) within the LSI groups and 38.3% (23/60) within CAU. This was non-significant between the groups (*p* = 0.845), see Table 2. 26/49 (53.1%) of the offspring were male and 23/49 (46.9%) were female within the LSI groups. For the CAU group this was 13/23 (56.5%) and 10/23 (43.5%), respectively. Mean time to conception after the start of the study was illustrated in a Kaplan–Meier curve in Figure 2, with 18.7 and 19.4 months within the LSI and CAU groups, respectively (*p* = 0.646). Although weight loss had a positive effect on the chance to become pregnant (see Figure 3), this was non-significant (β = −0.038 SE 0.028, *p* = 0.169).

A large proportion of the participants conceived spontaneously (42/72, 58.3%), with 55.1% (27/49) in the LSI groups and 65.2% (15/23) in the CAU group (*p* = 0.521). Median birth weight was 3350 g (2915–3760) and 3260 g (2810–3848) for the LSI and CAU groups respectively (*p* = 0.668), with a median gestational age at delivery of 39 weeks (37–40)) for the LSI group and 39 weeks (38–40) for the CAU group (*p* = 0.830).

### 3.2. Pregnancy and Neonatal Complications

Both (gestational) diabetes (LSI 8.2% (4/49) and CAU 21.7% (5/23); *p* = 0.133), and hypertensive disorder rates (LSI 8.2% (4/49) and CAU 13.0% (3/23); *p* = 0.673) during pregnancy were non-significantly different between the groups, see Table 2. Preterm birth accounted for 12.2% (6/49) in the LSI groups, and for 17.4 (4/23) in the CAU group (*p* = 0.716). NICU admission rates were 6.1% (3/49) in the LSI groups, and 13.0% (3/23) within the CAU group (*p* = 0.376). Both groups combined contained 5 cases with a congenital abnormality. From our own data we encountered one neonatal death in total due to a severe congenital disorder.

### 3.3. Prediction of Conception

**Figure 4:**
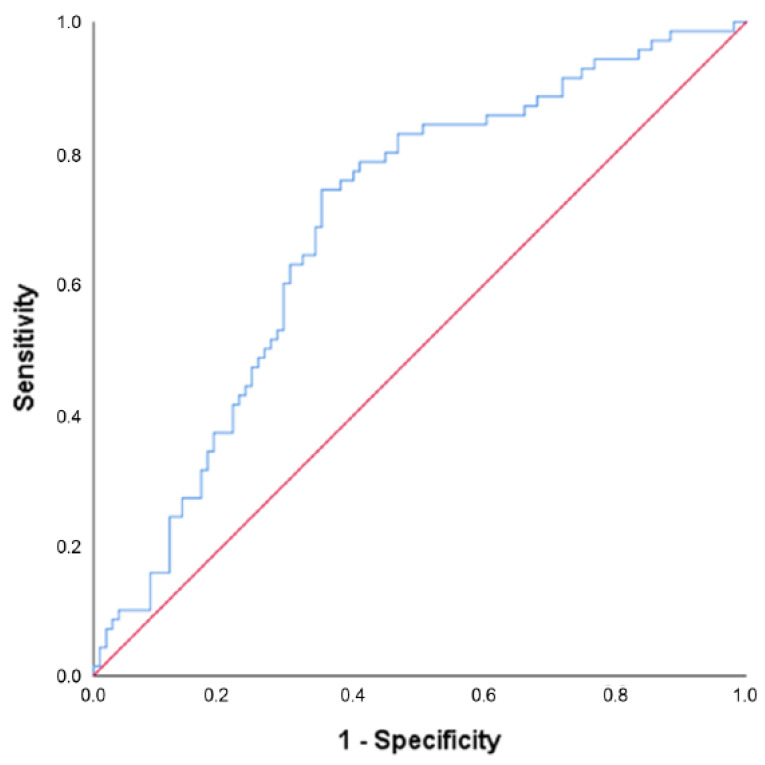
Receiver operating characteristic (ROC) curve for the model predicting conception within 24 months after the start of the intervention resulting in live birth. Note: Results are based on calculations by the Erasmus MC using non-public microdata from Statistics Netherlands. This final model included time attempting to conceive before the start of the study and insulin at baseline, area under the curve = 0.691 (*p* < 0.001).

Twelve potentially predicting baseline variables, further specified in Table 3, were identified and joined in a multivariate model. The stepwise elimination process resulted in a model in which time attempting to conceive before the start of the study (OR 0.984 (95% CI 0.972–0.997), *p* = 0.017) and insulin (OR 0.991 (95% CI 0.986–0.997), *p* = 0.003) at baseline both had a negative predictive value for conception resulting in live birth within 24 months after the start of the intervention (see Table 3). The ROC curve for the final model is displayed in Figure 4 with an area under the curve of 0.691 (*p* < 0.001).

### 3.4. Drop-Out Rate during Study Intervention Period

Finally, with the complete pregnancy data from the CBS and Dutch Perinatal registry we got more insight into participants who discontinued the intervention because of pregnancy or dropped out due to other causes. In previous publications we described a drop-out rate of 63.4% [19], which overestimated the number of true drop-outs as it included participants who dropped out due to pregnancy during the study period. With 28/123 pregnancies in the LSI group and 12/60 pregnancies in the CAU group there were a total of 40 (21.9%) pregnancies during the study intervention period, resulting in a true drop-out rate of 42.1%.

## 4. Discussion

This follow-up study from a randomized controlled one-year three-component lifestyle intervention reports on pregnancy outcomes based on data from the Dutch Perinatal registry. Conception rates and time to conception after the start of the study showed comparable non-significant results between the groups. It is worth mentioning that the majority of our population eventually conceived spontaneously. Pregnancy complications and outcomes were lower in the lifestyle intervention groups, and weight loss in general had a positive effect on the chance to conceive within 24 months after the start of the intervention. However, these findings were statistically non-significant. We also examined some predictors for pregnancy which resulted in a final model including baseline insulin level and time attempting to conceive before the start of the study.

Weight [19], emotional well-being [24], phenotypical characteristics [25], and metabolic health [26] all were shown to improve more in the LSI groups compared to CAU over the course of our study. It is believed that the pre-pregnancy optimization of these factors should improve reproductive and obstetric outcomes in women with PCOS as well as in their offspring [1]. Over the course of the study and follow-up period, women in all three groups got pregnant, either spontaneously or eventually aided by ART, as long as they reached their personal weight-loss goal at the end of the study. We observed coinciding increasing pregnancy rates and decreasing time to pregnancy after the start of the intervention in the lifestyle program. A similar trend was observed for pregnancy complications and adverse neonatal outcomes. It is interesting to see that the rates of pregnancy complications and adverse neonatal outcomes in the LSI group were, although still higher, more similar to the rates in the general Dutch population [27] when compared to the CAU group. However, the expected statistically significant differences were lacking. This could be explained by the fact that this study was powered on weight loss as the primary outcome [19], and not on pregnancy outcomes. Another explanation could be that the lifestyle intervention group was compared to care as usual, which also consisted of advice to lose weight. Although the amount of weight loss these women achieved was not as much as in the LSI group, this probably still had a positive influence on their chance to get pregnant.

Antenatal lifestyle interventions in the general population are associated with lower risks of adverse maternal and neonatal outcomes [28], which should be similar in women with PCOS. However, data on pregnancy outcomes reported from multi-component lifestyle interventions are lacking. A recent meta-analysis investigating the effect of lifestyle interventions in women with PCOS concluded that there were no studies which reported on live birth, miscarriage, or pregnancy [18]. However, Legro and colleagues did report on a preconception intervention (either 16 weeks of continuous oral contraceptive pills, lifestyle modification by low caloric diet, or both, followed by ovulation induction) in which live birth rates did not significantly differ between the groups [29]. The same group also demonstrated an improved live-birth rate as a benefit of delayed infertility treatment using clomiphene citrate (CC) when preceded by lifestyle modification with weight loss, compared to immediate treatment [30]. Furthermore, a few studies were performed on pregnancy outcomes in obese infertile women in general. These concluded that, although weight loss was achieved, lifestyle intervention preceding infertility treatment did not substantially affect live-birth rates [31,32,33]. However, we do have to keep in mind that success rates with fertility treatments are lower among obese infertile women when compared to normal-weight women [9,10], as well as the chance of natural conception [8]. Pregnancy and neonatal complications are also less common among non-obese women compared to obese women [34,35,36]. On top of this, women with PCOS have been found to be more prone to weight gain, which was most marked in those with unhealthy lifestyles [37]. Altogether, we would argue that recommending a lifestyle intervention in order to promote weight loss instead of immediately starting an infertility treatment in overweight or obese women with PCOS is the better choice. Moreover, a three-component lifestyle intervention aids in creating an overall healthier body composition in the metabolic, physical and mental domains which might as well result in a healthier pregnancy.

Based on our results, one could argue for the implementation of such a long-term and intensive lifestyle intervention for all women with PCOS, in order to improve fertility outcomes. Should we therefore look for other therapies to achieve even more weight loss, such as bariatric surgery? However, one should also keep in mind a treatment’s impact, side-effects and cost-effectiveness. Bariatric surgery is an invasive procedure, and will cause a delay in fertility treatment because it is undesirable to conceive during a period of rapid weight loss. Furthermore, pregnancy complications due to nutrient malabsorption after bariatric surgery are also possible [1,38]. Other less invasive options, such as the use of insulin sensitizers like metformin or thiazolidinediones, are proven to be beneficial for weight loss and the treatment of infertility in women with PCOS [39]. However, these drugs can cause gastro-intestinal side effects or even weight gain, which may reduce patient compliance [40]. Inositol as an insulin sensitizer is currently recognized as a possible candidate for a non-invasive low-cost addition to lifestyle therapy with lack of significant adverse effects, even in pregnancy [41,42,43]. Benefits such as improving the ovulation rate as well as hormonal and insulin sensitivity indexes have been demonstrated [44]. However, further evidence will be necessary to confirm the efficacy of inositol to improve pregnancies and live birth in women with PCOS [45]. Finally, the use of anti-obesity drugs such as glucagon-like peptie-1 receptor agonists are currently an emerging area of interest and could also be considered while developing treatment strategies for overweight women with PCOS. Although contraindicated during pregnancy, these anti-obesity drugs simultaneously improve insulin sensitivity, reduce cardiovascular disease risk, and show promising potential in achieving and maintaining weight loss [46].

Baseline insulin levels and time attempting to get pregnant before the start of the study both had a negative predictive value on the chance to conceive. The same factors along with other predictors were reported in studies predicting the chances for live birth after ovulation induction using anti-estrogens [10,47,48], or using gonadotrophins [49,50,51]. In addition, a large proportion in our population conceived spontaneously, which again may be driven by different baseline predictors. Overall, given this spontaneous conception rate, and knowing most of them had a long time to pregnancy before they entered the study, which is a negative predictor, these study results are encouraging and may support the advice of lifestyle changes prior to infertility treatment in this population.

A strength of this follow-up study is the utilization of pregnancy data from the Dutch Perinatal registry. Because of this, we were sure to collect data on all conceptions resulting in live birth within the given timeframe, and we could even report on pregnancy outcomes from women who were lost to follow-up from the RCT. On top of this, we could make a distinction between the “real drop-out” and women who became pregnant during the study but were lost to follow-up, which resulted in a lower overall study drop-out rate than previously reported for this RCT [19].

However, a limitation of data from the CBS is the absent knowledge on miscarriages and pregnancies that ended before 16 weeks of gestation. Nonetheless, the final desired end-goal of couples will be an uneventful pregnancy and the birth of a living child, which is therefore in our eyes the most important study outcome. Furthermore, one should keep in mind that not all women in our study ultimately received fertility treatment, which could also be seen as a limitation. Participants in our study only received fertility treatment after achieving their personal weight loss goal, whereas other studies generally treated all participants [29,31,32,33]. This may cause an underestimation of pregnancies in our study when compared to other study designs. However, we believe that it was more desirable for participants to primarily achieve their weight loss goal and a healthy lifestyle before the start of an infertility treatment in order to decrease the chance on any possible iatrogenic induced pregnancy complications associated with overweight or obesity [52].

## 5. Conclusions

In total, 39.3% of the women conceived within 24 months after the start of the study, of which 58.3% were spontaneous conceptions. Women in het LSI groups lost more weight compared to CAU based on our previous data; however, this follow-up study showed no significant differences in conception resulting in live birth rates between LSI and CAU. These results should be interpreted with caution, because the study was not powered for pregnancy outcomes.

## Figures and Tables

**Table 1:** Baseline characteristics.

	Lifestyle Intervention (SMS+ and SMS−) n = 123	Care as Usual n = 60
	n (%)	n (%)
Nulliparous	94 (77.7)	44 (75.9)
Smoking	24 (19.7)	14 (23.7)
Alcohol consumption	27 (22.1)	19 (32.2)
Ethnicity		
Northern European	52 (42.6)	24 (40.0)
Mediterranean	18 (14.8)	12 (20.0)
Hindustani	15 (12.3)	6 (10.0)
African	27 (22.1)	17 (28.3)
Asian	6 (4.9)	0 (0.0)
Other	4 (3.3)	1 (1.7)
Education		
Low	10 (8.3)	8 (14.3)
Intermediate	67 (55.4)	35 (62.5)
High	44 (36.4)	13 (23.2)
PCOS characteristics		
OD	118 (96.7)	57 (95.0)
HA	97 (80.2)	47 (78.3)
PCOM	118 (98.3)	59 (98.3)
Phenotype classification		
A (OD + HA + PCOM)	89 (74.8)	43 (71.7)
B (OD + HA)	2 (1.7)	1 (1.7)
C (HA + PCOM)	4 (3.4)	3 (5.0)
D (OD + PCOM)	24 (20.2)	13 (21.7)
		Median (IQR)
Age (year)	29 (26–32)	28 (26–32)
Weight (kg)	92 (83–105)	84 (79–97)
BMI (kg/m²)	33.6 (30.8–36.6)	30.6 (29.3–34.3)
Waist (cm)	101 (93–107)	96 (89–109)
Age of menarche (year)	12 (12–14)	12 (11–13)
Time attempting to conceive (months)	24 (15–38)	23 (14–35)

Note: Values are displayed as numbers (percentage) or as medians (interquartile range). Time attempting to conceive includes the time before the start of the study. Abbreviations: SMS+; lifestyle intervention with SMS support, SMS−; lifestyle intervention without SMS support, OD; ovulatory dysfunction, HA; hyperandrogenism, PCOM; polycystic ovarian morphology, IQR = interquartile range, BMI = body mass index.

**Table 2:** Pregnancy outcomes within 24 months after the start of the intervention.

	Lifestyle Intervention (SMS+ and SMS−)	Care as Usual		Total
	n (%)	n (%)	*p*	n (%)
Conception resulting in live birth	49/123 (39.8)	23/60 (38.3)	0.845	72/183 (39.3)
Stillbirth (ante partum)	-	-	-	3/75 (4.0)
Mode of conception				
Spontaneous	27/49 (55.1)	15/23 (65.2)		42/72 (58.3)
After ART	16/49 (32.7)	7/23 (30.4)		23/72 (31.9)
Unknown	6/49 (12.2)	1/23 (4.3)	0.521	7/72 (9.7)
Method of delivery				
Vaginal birth	25/49 (51.0)	11/23 (47.8)		36/72 (50.0)
Instrument-assisted/caesarean section	22/49 (44.9)	12/23 (52.2)		34/72 (47.2)
Unknown	2/49 (4.1)	0/23 (0.0)	0.564	2/72 (2.8)
Pregnancy complications				
(gestational) diabetes	4/49 (8.2)	5/23 (21.7)	0.133	9/72 (12.5)
Hypertensive disorders	4/49 (8.2)	3/23 (13.0)	0.673	7/72 (9.7)
Preterm birth	6/49 (12.2)	4/23 (17.4)	0.716	10/72 (13.9)
Adverse postpartum outcomes				
Hemorrhage	-	-	-	5/72 (6.9)
Adverse neonatal outcomes				
Apgar score < 7 after 5 min	-	-	-	3/72 (4.2)
NICU admission	3/49 (6.1)	3/23 (13.0)	0.376	6/72 (8.3)
Small for gestational age	6/49 (12.2)	4/23 (17.4)	0.716	10/72 (13.9)
Large for gestational age	5/49 (10.6)	4/23 (17.4)	0.452	9/72 (12.5)
Congenital abnormalities	-	-	-	5/72 (6.9)
	Median (IQR)	Median (IQR)		
Birth weight (grams)	3350 (2915–3760)	3260 (2790–3870)	0.668	
Birth weight (percentile)	64 (24–83)	69 (22–86)	0.817	
Gestational age at delivery (days)	276 (264–283)	276 (267–283)	0.633	
Apgar 5 min	10 (9–10)	10 (9–10)	0.734	

Note: Results are based on calculations by the Erasmus MC using non-public microdata from Statistics Netherlands. Values are displayed as number/total (percentage) or as medians (interquartile range). Differences were tested with the use of the X^2^ test or the Fishers exact test for categorical outcomes and with the use of the Mann–Whitney U test for continuous outcomes. There were no significant differences between the groups. Abbreviations: SMS+; lifestyle intervention with SMS support, SMS−; lifestyle intervention without SMS support, ART; assisted reproductive technology, NICU; neonatal intensive care unit, IQR = Interquartile range.

**Table 3:** Determinants of conception within 24 months after the start of the intervention.

Univariate Model	OR (95% CI)	*p*-Value
Age	0.939 (0.875–1.007)	0.078
Body mass index	0.877 (0.776–0.991)	0.035
Modified Ferriman–Gallwey score	0.959 (0.901–1.021)	0.191
Waist circumference	0.967 (0.930–1.006)	0.094
Time attempting to conceive	0.984 (0.971–0.997)	0.014
Androstenedione	0.906 (0.805–1.021)	0.105
Free androgen index	0.919 (0.852–0.992)	0.030
Glucose	0.564 (0.310–1.023)	0.060
Insulin	0.992 (0.986–0.997)	0.002
Sex hormone-binding globulin	1.020 (1.000–1.040)	0.049
Mean ovarian volume	0.925 (0.846–1.013)	0.091
Amenorrhea	0.535 (0.223–1.287)	0.163
Multivariate model	OR (95% CI)	*p*-value
Time attempting to conceive	0.984 (0.972–0.997)	0.017
Insulin	0.991 (0.986–0.997)	0.003

Note: Results are based on calculations by the Erasmus MC using non-public microdata from Statistics Netherlands. Logistic regression analyses, values are displayed as odds ratio (95% confidence interval), all model were corrected for baseline weight. Abbreviations: OR; odds ratio, CI; confidence interval.

## Data Availability

Parts of the data presented in this study are available on request from the corresponding author. The data are not publicly available due to participant privacy reasons. Restrictions apply to the availability of the CBS/Perined data. Data was obtained from CBS/Perined and are only available from the authors with the permission of CBS/Perined.
